# Quality of Life and Dependence Degree of Chronic Patients in a Chronicity Care Model

**DOI:** 10.3390/healthcare8030293

**Published:** 2020-08-24

**Authors:** Jesús Molina-Mula, Angélica Miguélez-Chamorro, Joana María Taltavull-Aparicio, Jerónima Miralles-Xamena, María del Carmen Ortego-Mate

**Affiliations:** 1 Department of Nursing and Physiotherapy, University of the Balearic Islands, 07122 Palma, Spain; 2 Chronicity Care, Sociosanitary Coordination and Rare Diseases in the Regional Health Service of the Balearic Islands, 07003 Palma, Spain; angelica.miguelez@ibsalut.es; 3 Primary Health Care Service in the Majorca Island, Balearic Regional Health Service, 07003 Palma, Spain; jtaltavull@ibsalut.caib.es; 4 Technical Office, Primary Care Service, Scientific Committee, J. Briggs Institute, 07003 Palma, Spain; jmiralles@ibsalut.caib.es; 5 Department of Nursing, University of Cantabria, 39008 Santander, Spain; carmen.ortego@unican.es

**Keywords:** chronic diseases, quality of life elderly, frail elderly, multiple chronic conditions, nursing care

## Abstract

Background: The complex chronic patient is a person with one or several long-term diseases, the clinical management of which are considered difficult and related to cognitive or functional impairment. The chronicity care model deeply affects the quality of life and degree of dependence. Objectives: The objective of this study was to analyse the perceived quality of life and dependence degree in complex chronic patients within a chronicity care model in the Autonomous Communities of Cantabria and the Balearic Islands (Spain). Design: This was a multicentred, transversal, descriptive, and observational study on a cohort of 206 chronic patients included in a chronicity care program. Methods: Patients’ sociodemographic variables, integral valuation, nurse follow-up records, nursing outcomes classification (NOC)/nursing interventions classification (NIC), nurse diagnoses, and hospitalization data were analysed. A descriptive analysis of all data was carried out. The bivariate analysis assessed the relation between covariables and the overall scoring in European Quality of Life Scale (EuroQuol-5D), Barthel, Braden, and Chronic Patient eXperience Assessment Instrument (IEXPAC in the Spanish abbreviation). A multivariate linear regression analysis was conducted. Results: The mean age was 79.4 years (standard deviation (SD) = 9.12; range: 39–94). A percentage of 79.3% of the study population shows functional impairment in one or more activities of daily life. A percentage of 83.3% of patients showed a physical dependence. There is a significant relationship between the gender and kinship degree of the caregiver (χ^2^ = 18.2; *p* = 0.001). An overall mean score of 55.38 points in EuroQuol-5D was obtained, along with a 36.87-point satisfaction with the care given in IEXPAC. The overall score correlated positively and significantly with Barthel, Braden, and IEXPAC. The dependence levels improved slightly in the observed patients, which was a very significant outcome in statistical terms (t = 2.08; *p* = 0.039). A percentage of 66% (R^2^ = 0.66) of the score variability at the Barthel index could be predicted from Braden scale scoring. Conclusions: Dependence is not only affected by the related pathology, but also by the effect on mobility and daily-life activities, which cause a worse perception of the quality of life. The health-care model based on the case management nurse is having positive effects, especially on dependence and patients with ulcer issues.

## 1. Introduction

The prevalence increase of chronic diseases in all stages of life, together with an uneven distribution of these within the general population, make a populational approach necessary to deal with this situation in full. A great part of the social and human impact caused every year by deaths related to chronic diseases could be avoided by means of well-understood, feasible, and profitable interventions [1].

Both social and health systems face one of the highest aging and chronicity rates in all of Europe, with an increasing trend on the rise. A percentage of 91.3% of the mortality rate and 86% of lost life years may be attributed to chronic diseases [2].

Ageing refers to a decreased functional ability; this does not automatically imply illness, yet it does imply more vulnerability to changes and to unfavourable environments. As people become older, the acute processes weigh less on morbidity and mortality and the chronic processes take the lead [3,4]. 

Complexity in people with chronic diseases, from the patients’ and caregivers’ point of view, is related to loss of functionality and spiraling visits to health services, which too frequently are fragmented and uncoordinated. The patients and relatives often do not know what to do and feel overwhelmed and confused by the maze of persons, environments, and interventions, to which we have to add up the existence of socioeconomic determinants, multiplying the complexity perception [5,6].

Ageing-associated multimorbidity and polymedication lead to disability and dependence [3], which, influenced by social issues and a healthcare service focused on acute pathology, end up in complex and advanced chronicity. The system’s inability to give an effective response to the people and their caregivers under distress from just one single social or medical resource adds even more complexity to the process and favours impairment by frailer persons [7].

Multimorbidity refers to the presence of more than one chronic disease, and a patient is labelled as polypathological when he or she suffers chronic diseases that are included in two or more predefined different categories. These pathologies are in general equally as complex and with a similar destabilization potential, existing operational difficulties, and reciprocal interactions [8]. In addition, the prevalence of multimorbidity and polypathology increases with age [5,6].

In recent years, the concepts of clinical complexity, complex chronic patients, and advanced chronic patients have been introduced. This patient profile makes up between 3% and 5% of the population and consumes a large percentage of resources since they are responsible for 20% to 40% of hospital admissions. A percentage of 40% are admitted three times a year or more, and 13% are five times or more [9,10].

A complex chronic patient is defined as a person with one or more long-term diseases, the clinical management of which are perceived as complicated by the reference health professionals, and which are associated with functional and/or cognitive impairment, requiring the orderly use of several health and social services. This definition, unlike the stratification systems, is based on three fundamental aspects helping to identify sick people with complex needs: a characteristic complexity clinical profile (multimorbidity, polypharmacy, decompensations, multiple admissions, etc.), a subjective professional judgement, and an improvement potential benefit, both individual and as a care team in a determined territorial context [9,11].

Dealing with this patient and the family perspective, case management as an advanced care practice has constituted a basic strategy in complex chronicity care all around the world, but it has not been enough. To constitute an integral care model guaranteeing multi-professional, coordinated attention based on the best evidence, it is paramount to launch groundbreaking actions in several domains in a synergic way on the same territory and population [12].

The functional impairment of elderly people during hospital stays is a serious problem in today’s health-care model. This impairment has serious consequences in terms of dependence, institutionalizing, a large amount of resources, and mortality. In three-month-tracking studies, 40% of people over 65 showed impairment in some instrumental activity of daily living (IADL) and 19% in basic activities of daily living (BADL) [13]. Zisberg, Shadmi, Sinoff, et al. [14] describe a 46% functional impairment secondary to overall hospitalization in over-70s who were admitted in internal medicine.

The National Strategy for Approaching Chronicity in the Spanish National Health System [3] and even the World Health Organisation [1] resoundingly affirm that primary health care (PHC) should be the focus of caregiving to people with chronic diseases. Safeguarding planned and proactive home care to people is more effective and efficient than institutionalized care [15].

To improve clinical effectiveness in the current context of chronicity and frailty and to reduce the overdiagnosis and medical overaction, the PHC must be strengthened [16,17]. Specific intervention programmes within the community that guarantee personalized and on-going care do reduce the amount of hospital admissions and average stays [18]. 

The Kaiser Permanente is a care model with a population approach based on clinical leadership and integrated care. Solving the capability in primary care settings is highly promoted and a strong emphasis is put on diminishing hospitalizations, which are regarded as a “system flaw” [19,20].

Mármol López, Miguel-Montoya, Montejano-Lozoya, et al. [21], in a systematic review in 2018, evaluate the impact of the nurse intervention in chronicity care in Spain, concluding that interventions carried out by case-management nurses (CMNs) yielded a more effective and efficient approach for people burdened with complex chronicity than those carried out following the traditional method.

La Berre, Maimon, Souria, et al. [22], in another systematic review in 2017, analysed the clinical effectiveness in transitional care management programs aimed at patients over 65 with polypathology or deemed geriatric, frail, or meeting the polypharmaceutical condition. A statistically significant reduction in the mortality rate was evinced at 3, 6, 12 and 18 months after discharge, and a reduction in hospital admissions after these 3, 6, 12 or 18 months was observed without noticeable differences in quality of life. The care-management nurse, responsible for coordination and care continuum, had played a key role.

Given the importance of launching a care model that is adapted to the complex care needs of people suffering multimorbidity and dependence, a comparative study between two regions in Spain is presented here. These have different care models, nevertheless sharing the same goal regarding the chronic complex patient, that is, aiming at improving their quality of life, reducing hospital admissions, and promoting self-care.

## 2. Materials and Methods 

### 2.1. Aim

The aim was to analyse the quality of life and the dependence degree of advanced and complex chronic patients in a chronicity care model in the Spanish Autonomous Communities of Cantabria and the Balearic Islands.

### 2.2. Design

This is a multicentred, cross-sectional, descriptive and observational study aimed at patients with complex chronicity residing in two health-care sectors in the aforementioned Spanish regions, i.e., Cantabria and the Balearic Islands.

### 2.3. Sample/Participants

From the population data, a 20% prevalence, an accuracy of +/−5 percentage units, a 95% level of trust, and a 35% replacement rate were adopted. A total of 103 patients from each region (Mallorca and Cantabria) were labelled chronic according to each region’s stratification system. For sample size determination, the Granmo Programme 7.12 version (URLEC Consortium, Barcelona, Spain) was used. The sampling type was intentional due to the difficulty of trying to reach these patients.

### 2.4. Cohort Description

To establish the research question, the scientific literature related to the phenomenon under study was reviewed, where the different experiences and perceptions of the patients were analyzed. Subsequently, professionals who cared for chronic patients were consulted, and the questions were adapted to the preferences of the patients.

For the selection of patients, the following inclusion criteria were established:

- Patients were included in the polypathological patient program and labelled polypathological chronic by the Primary Health-Care Service in Santander (Cantabria, Northern Spain) and patients classified as Chronic Complex or Advanced Chronic by the Primary Health-Care Service on the Island of Majorca (Balearic Islands).
- Patients lacked any cognitive impairment (assessed through the Minimental questionnaire with a score higher than 24).
- Patients were over 18 years old.
- Patients consented to taking part in the study.

The patients were contacted through the chronic care nurses of each service and asked to participate in the study, after they were given information about it. The patients reviewed the data collection notebooks to provide their opinion on suitability and difficulty in responding. In addition, the contributions made by patients of what information they considered relevant were taken into account.

The results of the studies were specifically disseminated among the participants, through information sessions where patients described their experiences to health professionals in chronic care.

From the research team, our thanks are transmitted to all the patients and families who participated, as well as to the health professionals who care for this type of patient.

### 2.5. Patient and Public Involvement

While the study was being carried out, the selected patients were included for evaluation in the chronicity care programs that are specific to each region and consist in (a) a previous visible identification for all professional staff, (b) integral functional, cognitive, and social assessment, (c) adaptation of the therapeutic plan, (d) a specific care route in the case of requiring hospital admission, and (e) coordination through case management between the hospital and the primary health service and between the health-care system and the social services.

### 2.6. Study Variables

Based on measurements known as adjusted morbidity groups (AMG), patients with an individual complexity over the 95th percentile, which is the stratum of greatest risk and complexity, constituted 3–5% of the population [23].

The study variables were classified into two groups: those suiting the chronic patient and those suiting the caregivers.

### 2.7. Data Collection

A notebook for the data collection was elaborated. It contains the sociodemographic variables, full assessments, nursing follow-up records, nursing outcomes classification/nursing interventions classification (NOC-NIC) nursing diagnoses, and hospitalization data, if available. Variables were as follows:

- sociodemographic features;
- performance in activities of daily living according to the Barthel index [24];
- the patient’s experience in the health-care system and with the health professionals through the IEXPAC (Fundación Vasca de Innovación e Investigación Sanitarias) [25];
- the health-related quality of life of the patient through the European Quality of Life Scale (EuroQuol-5D-5L) [26];
- the Braden scale to weigh the risk of suffering pressure ulcers (PU) [27];
- the need for palliatives through the NECPAL-CCOMS-ICO©TOOL Version 2.0 (ICO, Barcelona, Spain) [28].
- the abbreviated Minimental questionnaire to detect and assess cognitive impairment [29];
- the NOC outcome criteria through the North American Nursing Diagnosis Association (NANDA) nurse diagnosis and the NIC interventions;
- hospital admissions and stays, consultation, and care continuity.

The variables of hospital admissions and stays, consultations, and continuity of care were obtained through outcome indicators, provided by the epidemiological registers of the two health services involved, and included the number of home visits, the number of primary health system (PHS) consultations, the number of emergency service consultations, reasons for the consultations, the average stays in hospital, the number of admissions, the number of readmissions, and the number of follow-ups in the previous 12 months.

The variables of nursing care were obtained by means of the medical histories, collecting the standardized NANDA diagnoses, the NOC outcomes, and the NIC interventions scheduled on the selected patients.

### 2.8. Ethical Considerations

The Ethics Committee for Clinical Research of the Balearic Islands and Hospital Research’s Commissions authorized this study (IB3389/17PI). All participants received information, accepted, and signed informed consent forms. Data obtained were completely anonymized.

The codes of good practice in research, current legislation, and the research principles of the Declaration of Helsinki were respected. No conflict of interest existed between participants and researchers conducting the study.

### 2.9. Data Analysis

The statistical analysis was carried out with the statistical software SPSS (IBM, Armonk, NY, USA) Statistics 22 and the Excel Spreadsheet program. A bilateral contrast and a 95% trust level were adopted.

The normality of the distribution was estimated through the Kolmogorov–Smirnov test, and the variance homogeneity was analysed with the Levene test. The presence of atypical scores was searched by carrying out a sensitivity analysis with the aim of finding out how these scores affected the outcomes. As long as the normality postulates were fulfilled, parametric and non-parametric techniques were applied.

A univariate analysis of all data was conducted. The quantitative variables are described by the mean and the standard deviation and the qualitative variables through absolute and relative frequencies. A bivariate analysis was used to examine the association between covariables and overall scores in EuroQoL-5D, Barthel, Braden, and IEXPAC. After researching suppositions that might be related to the normal distribution of residuals and multicollinearity, a multivariate linear regression analysis was conducted utilising the backward elimination model with a *p*-value < 0.20.

### 2.10. Validity and Reliability (Rigour)

To ensure the internal and external validity of the study, the STROBE recommendations for observational and descriptive studies have been observed. The utilised tools conformed to the rigour and validation criteria, contrasted in a multitude of studies. The professionals who collected the data have been trained for a correct assessment of the scales, and extreme variations have been statistically corrected.

## 3. Results

The total of the collected sample amounts to 206 complex chronic patients (103 in Cantabria and 103 in the Balearics). The mean age was 79.4 years (SD = 9.12; range: 39–94). It should be highlighted that most of the participants had finished primary school, owned their homes, and had a monthly income under 1000 €. No significant differences were detected when the patient’s sociodemographic features were compared in both regions, neither by age (t = 1.60, *p* = 0.112), by gender (χ^2^ = 0.49, *p* = 0.485), by educational level (χ^2^ = 6.04, *p* = 0.110), by income level (χ^2^ = 0.015, *p* = 0.904), by previous occupation (χ^2^ = 11.38, *p* = 0.123), nor by living status (χ^2^ = 0.48, *p* = 0.519).

No cognitive impairment was detected, neither 12 months after the study completion (mean 31.03; SD = 4.17) nor at the beginning (mean 30.39; SD = 4.17). The slight decrease observed at the beginning was ultimately insignificant (t = 0.130; *p* = 0.897). There is a significant relationship between the gender and kinship degree of the caregiver (χ^2^ = 18.2; *p* = 0.001). By men, it is their spouse, and by women, one of their daughters.

In relation to the dependence profile, it turns out that 79.3% (*n* = 163) of the study population presents functional impairment in one or more activities of daily living. A percentage of 83.3% (*n* = 90) of the chronic patients presented a physical dependence, of which 77 were moderate (72%) followed by severe (15.9%; *n* = 17) or great dependence (8.4%, *n* = 9). A percentage of 69.6% (*n* = 126) lived with another dependent person in the same household. Of the 163 participants with a dependency profile, the mean Barthel index at the start of the study was 78.65 points (SD = 22.13), 19.01 (SD = 2.83) on the Braden scale, 36.87 (SD = 12.81) in the IEXPAC, 30.39 (SD = 2.86) in the Minimental, and 55.38 (SD = 1.18) in the visual analog scale of the EuroQol-5D-5L, with 1.42 hospital admissions of (SD = 1.66) a year on average.

To establish the profile of the dependent patient, the Barthel index, the Braden scale, and the Visual Analog Scale (EVA) were utilised. The dependence degree for the activities of daily living, measured through the Barthel index 12 months before the start of the study, scored 72.82 points (SD = 25.65), which shows that the participants were situated in a range of mild dependence (95–60 points according to the index). [25]

After evaluation at the beginning of the study, the aforementioned mean increased to 78.65 points (SD = 22.13), which shows that the dependence levels improved slightly by the observed patients, which was a very significant outcome in statistical terms (t = 2.08; *p* = 0.039). A percentage of 66% (R^2^ = 0.66) of the score variability at the Barthel index could be predicted from the Braden scale scoring, the IEXPAC scale, the number of nurse home visits, the number of case management nurse’s consultations, and the follow-ups via telephone (Table 1 and Table 2).

The risk of suffering pressure ulcers was estimated with the Braden scale. For the previous 12 months, the mean equaled 18.37 points (SD = 2.83), a low risk that may in fact require weekly monitoring. When the study was started, the scoring increased until reaching 19.01 (SD = 2.47), therefore decreasing the risk of being monitored if any changes arise. Nevertheless, this difference was ultimately insignificant (t = 0.51; *p* = 0.608). The Barthel and the Braden test scorings obtained an intense, positive, and significant correlation (rxy = 0.73; *p* = 0.000) (Table 1 and Table 2).

As expected, as age augmented the Barthel index score significantly diminished (rxy = 0.21; *p* = 0.003), it also augmented the Braden scale (rxy = 0.19; *p* = 0.009) and the Minimental (rxy = 0.19; *p* = 0.008), and the amount of primary care emergency service consultations increased (rxy = 0.15; *p* = 0.047) (Table 1 and Table 2).

Continuing with the dependence-related variables, the relationship between owning a home and a greater number of participants with physical dependence was verified (χ^2^ = 24.3; *p* < 0.000), but these participants were not receiving dependence subsidies (χ^2^ = 7.0; *p* = 0.008). The rest of the variables did not show significant associations.

The average monthly income determined the dependence type and level (χ^2^ = 20.5; *p* < 0.000). As expected, those more likely to receive financial support were people with an income under 500 €/month (41.1%; *n* = 23) and between 500 and 999 €/month (39.3%; *n* = 22) (χ^2^ = 9.5; df 3; *p* = 0.023). Non-attendance to day care centres was linked to all income ranges (χ^2^ = 8.0; df 3; *p* = 0.044). However, higher incomes were linked to greater satisfaction with the service received, determined by IEXPAC (χ^2^ = 22.1; *p* = 0.032), and to a lower perception of anxiety and depression, EuroQol-5D (χ^2^ = 29.9; *p* = 0.003).

With regard to the use of health-care services, persons with incomes from 500 € to 999 €/month were more frequent users of Primary Healthcare Nursing (PHN) consultations (χ^2^ = 130.0; *p* = 0.020) and telephone follow-ups (χ^2^ = 77.0; *p* = 0.02). No significant differences were detected in the rest of variables.

More than 51% (*n* = 91) of dependent persons living together with two or more dependent persons in the same household were not depending on any economic aid (χ^2^ = 17.8; *p* < 0.000).

A significant relationship was found between people not receiving home help and those having difficulty with self-care. It was also verified that fewer hours devoted to self-care was proportional to a greater difficulty with mobility.

In health-related quality of life (HRQoL), evaluated by means of EuroQol-5D, an overall mean score of 55.38 points (SD = 22.96) was obtained. The average satisfaction level, evaluated through IEXPAC, was 36.87 points (SD = 12.81). The overall score correlated with the Barthel index scorings (r = 0.23; *p* = 0.001), the Braden scale (r = 0.15; *p* = 0.040), and the IEXPAC in a positive and significant way (rxy = 0.27; *p* = 0.000) (Table 1).

The people who used to work in unqualified jobs (32.1%; *n* = 42) and in the service sector (32.8%; *n* = 43) presented a higher dependence according to the Barthel index, and their health-care experience appears to be worse after the IEXPAC (χ^2^ = 192,3; *p* = 0.002). They have less insight on quality of life according to EuroQoL-5D-5L in anxiety and depression (χ^2^ = 54.4; *p* = 0.002).

Comparing the five dimensions comprising EuroQol 5D-5L shows that problems appear usually in mobility and grooming. Even so, 45.7% (*n* = 91) show moderate or severe pain and unrest, and 26% (*n* = 52) show anxiety and depression issues, moderate or severe.

In reference to the use of health-care services, the most frequent visits were paid to the nurse, with a total of 3.274 visits to the nurse (mean 6.40; dt 15.40; *p* < 0.000), the PHC nurse (mean 9.42; dt 10.56; *p* < 0.000), and the case management nurse.

The emergency assistance average indicates that the attendance was 1.32 and 1.42 per patient at PHS and at hospital level, respectively, over the previous 12 months. In addition, nearly all patients were admitted in a hospital for longer than 2 days. People receiving home care spent fewer days at the hospital (r = −0.43; *p* = 0.002), and 51.8% (*n* = 27) stayed for longer than 5 days.

Most of the reasons for consultation were due to chronic diseases such as diabetes, cardiovascular conditions, arthritis, renal conditions, and respiratory problems, among others.

People who used to work at non-qualified jobs (32.1% − *n* = 42) and in the service sector (32.8% − *n* = 43) more frequently attended primary care nurse consultations (χ^2^ = 264.2; *p* = 0.016), doctor consultations (30.9% − *n* = 60 in both sectors; χ^2^ = 286.0; *p* < 0.000), and visited PHC emergency rooms (31.8%, *n* = 57, in the service sector; 27.9%, *n* = 50, in non-qualified positions; χ^2^ = 118.7; *p* = 0.008).

Since a greater amount of consultations are attended to by a nurse, in order to assess the nurse’s activity with complex chronic patients as thoroughly as possible, data regarding their main goals and interventions, data about the care plan availability, NANDA diagnoses, NOC aims, and NIC interventions were gathered.

Only 40.3% (*n* = 83) had a registered care plan, but only 33.5% (*n* = 69) had a nursing diagnosis. These diagnoses were in accordance with the kind of problems shown by chronic patients, “physical mobility impairment” being the most commonly mentioned nursing diagnosis, in spite of just 10.2% (*n* = 21) of the cases having actually been diagnosed. Moreover, fall prevention was the most usual NIC intervention. Therefore, this intervention was accompanied by the NOC of fall prevention behavior; needing help to prevent falls, 12.6% (*n* = 26) of the patients.

There is a relationship between the patient’s positive perception about the home care given after a hospital admission, measured by the IEXPAC scale, and the care plan schedule by the primary care nurse (χ^2^ = 20.5; *p* = 0.009). This care plan is also significantly related to patients without self-care issues (χ^2^ = 21.4; *p* = 0.006) or moderate problems in daily activities (χ^2^ = 22.8; *p* = 0.004) according to EuroQuol-5D-5L.

The nursing diagnoses and NOC aims are not linked to a perception of improvement in care quality according to the IEXPAC and the EuroQol-5D-5L scales. NOC aims, with a higher number of primary care nurse consultations, did show statistical significance (χ^2^ = 62.0; *p* < 0.000) and so did those of general practitioners (χ^2^ = 64.0; *p* < 0.000) and nursing NIC interventions (χ^2^ = 59.0; *p* = 0.000 and χ^2^ = 61.0; *p* < 0.000 respectively).

## 4. Discussion

Adult individuals with several chronic pathologies took part in this study. They came from well-differentiated health-care areas, both geographically and economically, in Spain. It became obvious that those with a lower educational and income level, with previously worse paid occupations, presented a greater dependence, had a worse health service experience, and had a low awareness of their quality of life, as has occurred in similar research [30]. In addition, they attended consultations from primary care nurses and general practitioners and used primary care emergency services more often, as Moral, Gascón and Abad show [31].

Multiple studies reflect that this relationship between low socioeconomic standing and vulnerability and dependence is recurring [11,32].

The women’s cohort was slightly greater in number than the men’s. However, although women usually live longer, they present a higher chronic disease prevalence and have a more negative perception of their health condition, as found in several other studies [33].

An increase in the number of persons aged 65 and over living in one-person households was found, and this is related to a higher life expectancy and consequently the likelihood of reaching old age alone [34,35,36]. In this study, 24.4% of participants lived in one-person households.

When comparing the functional impairment outcomes with those by Ibarrola et al. [33], it is clear that the population of this study is more dependent. In our study, people without dependence were 14.9%, versus 6% in Ibarrola. At the same time, people with great or severe dependence scoring under 60 in the Barthel index reached only 19.2% versus 54% in Ibarrola. These differences can be explained by the unequal number of Barthel index records, almost 67% in our study versus 93% in the other study.

When analysing the model repercussion in the use of care resources, we found that there were 1.32 visits to primary care emergency services per patient per year, and 1.42 visits to hospital emergency services. This datum is so different from other studies [31,33,37] that it gives us reason to suspect that the type of service being evaluated is not the same, since the record obtained by Ibarrola is a reduced emergency attendance rate per patient/year of 9.3 to 9.0, not detected in the control group.

In spite of that, in the present study, we cannot appraise the reduction in the strict sense, since we did not incorporate a control group, and no further follow-up was undertaken. What seems to take shape is the positive outcome of a scheduled and systematized chronicity care model for an efficient use of health-care resources [33,38,39].

As far as the care activity goes, the nurse is the health professional who receives more visits per patient/year, above all types of visits by other health professionals, in accordance with the strategy for the chronicity approach in the National Health System [3]. Authors have stressed the importance of the primary health-care nurse for assisting this population group, and chronic patients consider him/her a pivotal reference for improvement in their quality of life [38,39]. That is, they come to their designated nurse to clarify doubts, have their health status tracked, obtain advice on the approach to diversify daily life scenarios or scenarios experienced when receiving emergency service, and receive help in resource management. To sum up, the nurse ensures the continuity of care at all assistance levels.

As was foreseen, the care continuity was more valued through the IEXPAC by the patients experiencing an integrated care model with case management. All research that has assessed case management proves that the interventions undertaken by CMNs were ultimately more effective and efficient in the approach to helping people with complex chronicity than those carried out using the traditional method [21,40,41].

The outcome improvement can be explained through the intervention activated by the implementation of the new chronicity care model, which is focused on person needs, complication avoidance, and care continuity with case management led by advanced practice nurses.

The current organization of services, focused on the resolution of acute pathologies, favors an episodic attention to health problems with a curative approach, giving little value to preventive aspects, the perspective of care, and the responsibility people have for themselves. Furthermore, there is unwarranted variability in the care provided to patients with similar clinical characteristics, and interventions are carried out that do not add value in terms of improvement in health outcomes.

The lack of coordination between levels of health care (primary care vs. specialized care) and between the health and social systems is one of the main determining factors of inefficiency in the provision, development, and management of available resources and leads to services not being provided at the most appropriate level or by the most suitable professional.

The health system cannot offer only monitoring and discontinuous care to patients with chronic diseases, as this is generally linked to exacerbations or improvements of their pathologies. The approach to chronicity requires promoting work in interdisciplinary teams, formed by different professionals in health and social services involved in the care of these patients, who guarantee continuity of care with the maximum participation of the patient and their environment. 

The improvement of a health system organization in responding to the challenge of chronicity does not necessarily imply an increase in resources, but it does require adaptation and optimization in the use of the means already available. Thus, it is necessary to increase the responsibility of managers, professionals, and the general population.

### Limitations

The main limitation in this study is that the sample was not randomly selected. The difficulty of accessing patients and collecting their data for the research team led to the necessity of applying purposive sampling. Nevertheless, to minimize the possible bias, the team ensured that the main sociodemographic and health variables from both Cantabria and the Balearics were equivalent. Furthermore, patients were recruited by nursing professionals working in various PHC centres for the greatest possible heterogeneity. Likewise, previous to the sample selection, the nurses were trained in data collection, which was supervised via periodic meetings. Another limitation is the use of indirect methods, such as scales, as principal tools to assess dependence, health-related quality of life, and satisfaction with the care given. With the aim of reducing this bias, highly validated and utilised scales were used.

We must also point out that the lack of a unified register of the participants’ medical histories was a hindrance, especially concerning the dependence valuation scales, the care plans, and the NIC and NOC diagnoses.

## 5. Conclusions

Responding to the aims of the study undertaken, the perceived chronic patient satisfaction was conditioned by factors such as gender, the seriousness and degree of physical or psychical dependence, hours devoted to care, socioeconomic conditions, subsidies granted by the public administration, and care provided by professionals, especially the PHC nurse and the case manager.

The elicited outcomes in this study bring to light the fact that dependence is not only affected by the related pathologies, but also by how it affects mobility and daily activities; it generates a worse perception of quality of life. They exploited the convenience of the health-care model based on the case-management nurse, which is especially suitable for dependence or ulcer risk in patients.

These patients’ complexity, their demanding need of care and health, and their family and social resources imply the necessity of a population and community approach that influences the social health determinants and improves intersectoral coordination and the available resources—specifically, on population health interventions integrating strategies such as health education, health-promoting policies, and intersectoral mobilization and coordination. Likewise, it is clear that the role and function of nurses must be strengthened, while guiding their responsibility towards the role of the self-care educator, the case manager in patients with conditions of particular complexity, and the linking agent to improve transition between domains and care units.

## Figures and Tables

**Table 1:** Correlations among variables.

Variables		1	2	3	4	5	6	7	8	9	10	11	12	13	14	15	16	17
1	Age	1																
2	Number of dependent persons in the usual home	0.123	1															
3	Number of falls in the previous 12 months	0.094	0.034	1														
4	Barthel index score	−0.204 **	−0.207 **	−0.248 **	1													
5	Braden index score	−0.192 **	−0.146	−0.191 **	0.734 **	1												
6	IEXPAC scale score	0.080	−0.052	0.016	0.051	−0.128	1											
7	Minimental questionnaire score	−0.188 **	0.044	0.094	0.154 *	0.199 **	−0.025	1										
8	EuroQol-5D-5L Analogical visual scale score	0.047	−0.189 *	−0.023	0.227 **	0.151 *	0.265 **	0.045	1									
9	Number of nurse home visits	0.118	0.177 *	0.028	−0.262 **	−0.095	0.063	−0.024	−0.010	1								
10	Number of doctor home visits	0.060	0.061	0.009	−0.040	−0.033	0.137	−0.088	0.025	0.053	1							
11	Number of primary care nurse visits	−0.103	−0.110	−0.055	0.159 *	0.130	0.041	−0.005	−0.082	−0.162 *	0.528 **	1						
12	Number of general practitioner visits	−0.021	−0.067	−0.101	0.119	0.127	−0.092	0.107	−0.043	−0.112	−0.076	0.310 **	1					
13	Number of Case Managing Nurse consultations	0.074	0.012	−0.030	−0.247 **	−0.140	−0.026	−0.205 **	0.042	−0.029	−0.062	−0.026	0.005	1				
14	Telephone follow-ups.	0.124	0.178 *	−0.074	−0.288 **	−0.121	−0.107	−0.146	0.054	0.190 **	0.093	−0.012	0.130	0.441 **	1			
15	Number of PHC emergency service consultations	0.146 *	0.138	0.006	−0.329 **	−0.266 **	0.129	−0.063	−0.100	0.040	0.062	−0.006	0.140	0.211 **	0.160 *	1		
16	Number of hospital emergency consultations	0.110	−0.049	0.097	−0.224 **	−0.211 **	0.004	−0.038	0.045	−0.021	0.126	0.094	−0.031	0.221 **	−0.025	0.259 **	1	
17	Number of hospital admissions	−0.094	0.110	0.029	−0.074	−0.046	−0.071	−0.279 *	0.067	0.143	0.072	−0.004	−0.045	0.394 **	0.342 **	0.204	0.087	1

** The correlation is significant at level 0.01 (2 tales). * The correlation is significant at level 0.05 (2 tails).

**Table 2:** Multivariant linear regression models for scores in IEXPAC, Barthel, Braden, and EuroQuol.

Dependent Variable	*Prediction Variable*	*B*	*SE*	*β*	*t*	*p*
*IEXPAC*	(Constant)	52.48	8.11		6.47	0.000
	Barthel index score	0.25	0.08	0.42	3.27	0.001
	Braden scale score	−2.36	0.59	−0.47	−3.99	0.000
	Analogical visual scale. EuroQol-5D-5L	0.19	0.05	0.33	4.10	0.000
	Number of nurse home visits	0.12	0.06	0.16	1.91	0.059
	Number of primary care nurse consultations	0.12	0.08	0.13	1.59	0.115
	Number of general practitioner consultations	−0.21	0.16	−0.11	−1.32	0.190
	Number of case managing nurse consultations	0.70	0.45	0.14	1.56	0.120
	Number of telephone follow-ups	−0.42	0.27	−0.14	−1.54	0.127
	Model statistics: *R*^2^ = *0.26* Adjusted *R*^2^ = 0.21; *F* (8.125) = 5.47 **					
*Barthel*	(Constant)	−47.56	9.66		−4.93	0.000
	Braden scale score	6.12	0.44	0.72	13.80	0.000
	IEXPAC scale score	0.34	0.09	0.20	3.84	0.000
	Number of nurse home visits	−0.24	0.07	−0.19	−3.54	0.001
	Number of case managing nurse consultations	−1.10	0.50	−0.13	−2.19	0.031
	Number of telephone follow-ups	−0.47	0.30	−0.09	−1.57	0.120
	Model statistic: *R*^2^ = 0.66; Adjusted *R*^2^ = 0.65; *F* (5.128) = 50.41 **					
*Braden*	(Constant)	9.02	1.88		4.80	0.000
	IEXPAC scale score	−0.05	0.01	−0.24	−4.16	0.000
	Barthel index score	0.09	0.01	0.79	13.07	0.000
	Minimental questionnaire score	0.12	0.06	0.12	2.11	0.037
	Analogical visual scale. EuroQol-5D-5L	0.01	0.01	0.09	1.52	0.132
	Number of nurse home visits	0.02	0.01	0.13	2.27	0.025
	Number of case managing nurse consultations	0.10	0.06	0.10	1.71	0.090
	Model statistic: *R*^2^ = 0.63; Adjusted *R*^2^ = 0.61; *F* (6.127) = 36.23 **					
*Escala visual analógica. EuroQol-5D*	(Constant)	−33.93	23.03		−1.47	0.143
	Age	0.34	0.20	0.14	1.73	0.087
	IEXPAC scale score	0.60	0.14	0.36	4.39	0.000
	Braden scale score	2.09	0.69	0.25	3.02	0.003
	Number of telephone follow-ups	0.54	0.41	0.11	1.32	0.190
	Model statistic: *R*^2^ = 0.18; Adjusted *R*^2^ = 0.15; *F* (4.129) = 7.05 **					

*B* = unstandardized B coefficient, *SE* = standard error, *β* = standardized beta coefficient, *t* = test statistic, *R*^2^ = coefficient of determination, *p* = probability value, ** *p* < 0.001.
